# Predictive value of cardiac magnetic resonance right ventricular longitudinal strain in patients with suspected myocarditis

**DOI:** 10.1186/s12968-023-00957-6

**Published:** 2023-08-17

**Authors:** Benedikt Bernhard, Giulin Tanner, Davide Garachemani, Aaron Schnyder, Kady Fischer, Adrian T. Huber, Yasaman Safarkhanlo, Anselm W. Stark, Dominik P. Guensch, Jonathan Schütze, Simon Greulich, Jessica A. M. Bastiaansen, Maryam Pavlicek-Bahlo, Dominik C. Benz, Raymond Y. Kwong, Christoph Gräni

**Affiliations:** 1 Department of Cardiology, Inselspital, Bern University Hospital, University of Bern, Freiburgstrasse 18, 3010 Bern, Switzerland; 2 Department of Anesthesiology and Pain Medicine, Inselspital, University Hospital Bern, University of Bern, Bern, Switzerland; 3 Department of Diagnostic, Interventional and Pediatric Radiology, Inselspital, Bern University Hospital, University of Bern, Bern, Switzerland; 4 Department of Cardiology and Angiology, University of Tübingen, Tübingen, Germany; 5 Cardiovascular Division, Department of Medicine, Brigham and Women’s Hospital, Boston, MA USA

**Keywords:** Myocarditis, Right ventricle, Cardiac magnetic resonance imaging, Feature tracking, Right ventricular global longitudinal strain, Heart failure hospitalizations

## Abstract

**Background:**

Recent evidence underlined the importance of right (RV) involvement in suspected myocarditis. We aim to analyze the possible incremental prognostic value from RV global longitudinal strain (GLS) by CMR.

**Methods:**

Patients referred for CMR, meeting clinical criteria for suspected myocarditis and no other cardiomyopathy were enrolled in a dual-center register cohort study. Ejection fraction (EF), GLS and tissue characteristics were assessed in both ventricles to assess their association to first major adverse cardiovascular events (MACE) including hospitalization for heart failure (HF), ventricular tachycardia (VT), recurrent myocarditis and death.

**Results:**

Among 659 patients (62.8% male; 48.1 ± 16.1 years), RV GLS was impaired (> − 15.4%) in 144 (21.9%) individuals, of whom 76 (58%), 108 (77.1%), 27 (18.8%) and 40 (32.8%) had impaired right ventricular ejection fraction (RVEF), impaired left ventricular ejection fraction (LVEF), RV late gadolinium enhancement (LGE) or RV edema, respectively. After a median observation time of 3.7 years, 45 (6.8%) patients were hospitalized for HF, 42 (6.4%) patients died, 33 (5%) developed VT and 16 (2.4%) had recurrent myocarditis. Impaired RV GLS was associated with MACE (HR = 1.07, 95% CI 1.04–1.10; p < 0.001), HF hospitalization (HR = 1.17, 95% CI 1.12–1.23; p < 0.001), and death (HR = 1.07, 95% CI 1.02–1.12; p = 0.004), but not with VT and recurrent myocarditis in univariate analysis. RV GLS lost its association with outcomes, when adjusted for RVEF, LVEF, LV GLS and LV LGE extent.

**Conclusion:**

RV strain is associated with MACE, HF hospitalization and death but has neither independent nor incremental prognostic value after adjustment for RV and LV function and tissue characteristics. Therefore, assessing RV GLS in the setting of myocarditis has only limited value.

**Supplementary Information:**

The online version contains supplementary material available at 10.1186/s12968-023-00957-6.

## Background

Myocarditis is an important cause of heart failure (HF) that has recently gained attention due to the increasing number of patients undergoing high-sensitive noninvasive imaging modalities for myocardial tissue characterization such as cardiac magnetic resonance imaging (CMR). Both, clinical presentation and long-term outcomes of patients with myocarditis vary and can range from asymptomatic, mild and benign course to patients that suffer from a large spectrum of adverse cardiac events including HF with need for hospitalizations, ventricular tachycardia (VT), recurrent myocarditis, and death [1–3]. This underlines the unmet need for adequate risk-stratification tools that can support the physician in guiding patient management within this clinical setting. Based on right ventricular (RV) ejection fraction (EF), recent data underscore the important prognostic role of RV involvement in suspected myocarditis [4, 5]. However, RVEF does not fully characterize RV myocardial energetics as it is influenced by ventricular preload and afterload across the cardiac cycle and other factors [6, 7]. Feature tracking based assessment of global longitudinal strain (GLS) by CMR is a modern technique that has demonstrated high reproducibility [8], and incremental prognostic value by evaluating the left ventricle (LV) of patients with acute myocarditis [9], but has never been investigated in the RV in this setting and is not routinely performed in clinical practice. We tested the hypothesis that RV GLS might provide incremental prognostic value for clinical outcomes after suspected myocarditis over RVEF alone and other established predictors such as LVEF [10] and the presence of late gadolinium enhancement (LGE) [11–13].

## Methods

### Patients and design

**Fig. 1:**
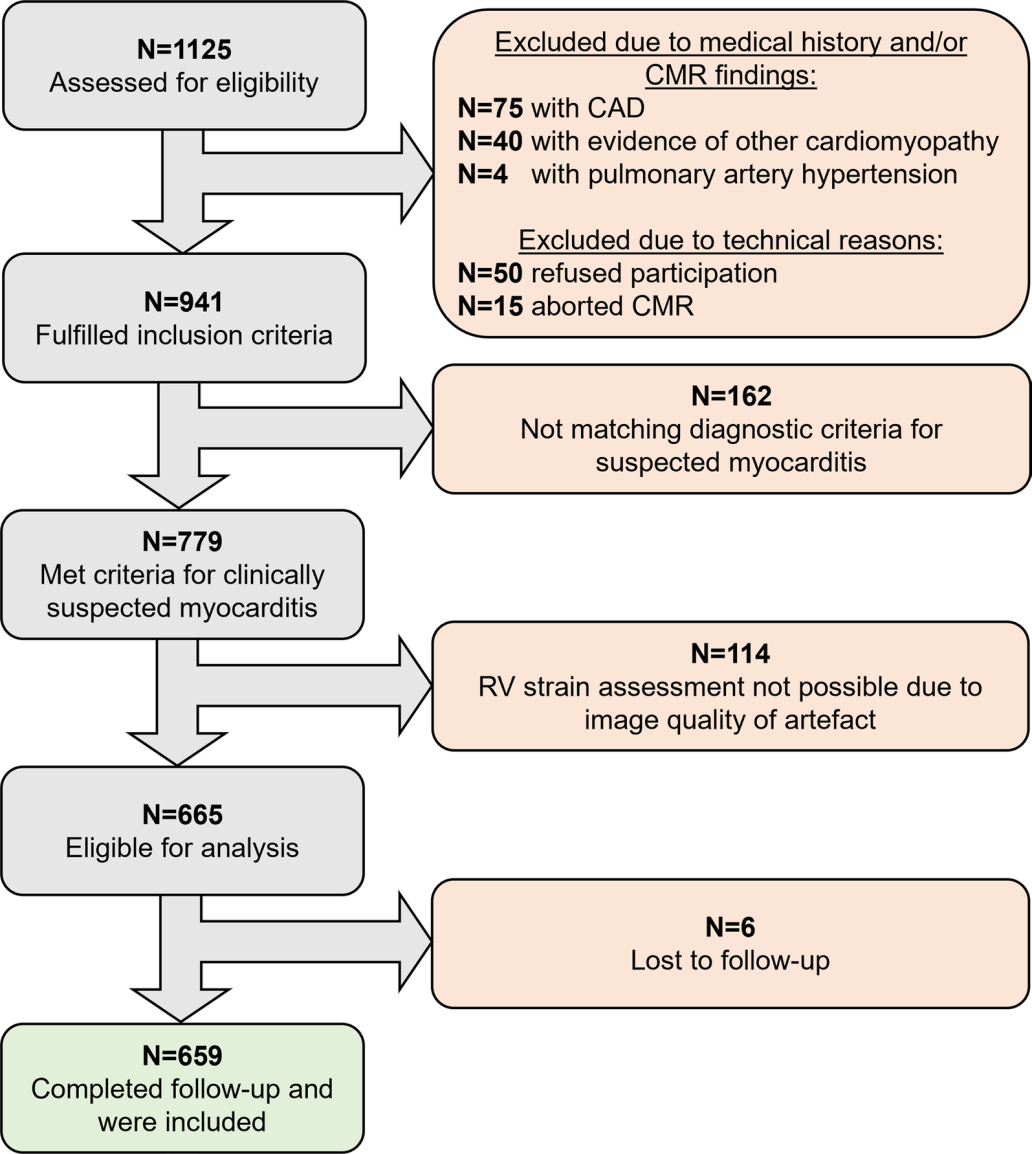
Study cohort. CMR: cardiac magnetic resonance. Criteria for clinically suspected myocarditis was defined according to Caforio et al. [1]

The design of the ‘Inflammatory Cardiomyopathy Bern Registry’ (FlamBeR) (NCT04774549) and the ‘CMR Features in Patients With Suspected Myocarditis’ (CMRMyo) (NCT03470571) registry was previously described [5]. In brief, consecutive patients who were referred for CMR at Inselspital, University Hospital Bern, Switzerland and Brigham and Women’s Hospital, Harvard Medical School Boston, Massachusetts, USA due to clinically suspected myocarditis between 2002 and January 2019 were enrolled in this register cohort study. Clinically suspected myocarditis was defined according to the position statement of the European Society of Cardiology (ESC) Working Group on Myocardial and Pericardial Diseases including clinical (acute chest pain, dyspnea or fatigue, palpitation, arrhythmia, syncope or sudden cardiac death or unexplained cardiogenic shock) and diagnostic criteria (positive ECG, elevated troponin, functional or structural abnormalities on cardiac imaging such as the presence of edema or LGE of classical myocarditis pattern in CMR) [1]. Myocarditis is clinically suspected if the patient meets either (a) ≥ 1 clinical criteria and ≥ 1 diagnostic criteria or (b) ≥ 2 diagnostic criteria [1]. Patients with evidence of coronary artery disease (medical history, anatomical or functional imaging findings including suspected ischemia by any imaging modality or endocardial LGE by CMR consistent with infarction, or invasive coronary angiography), or evidence of pre-existing pulmonary artery hypertension, pulmonary embolism or severe pulmonary disease, hypertrophic cardiomyopathy, arrhythmogenic cardiomyopathy, cardiac amyloidosis, Takotsubo cardiomyopathy, ventricular non-compaction, persistent severe valve disease, prior heart transplantation or prior cardiovascular surgery were excluded (Fig. 1). The primary outcome of first major adverse cardiovascular events (MACE) included HF hospitalization, documented VT lasting more than 30 s, recurrent myocarditis according to above mentioned criteria [1], and death. HF hospitalization was defined according to the ESC guideline definition of HF events [14]. The individual components of MACE were considered as secondary endpoints. Outcome was assessed by the patient’s chart review, documentations from referring physicians, hospital discharge summaries and by telephone interviews if there was a lack of documentation. Censoring events were the last available follow-up or the patient’s death. Both registries were approved by the local ethics committees and the study was conducted in accordance with the Declaration of Helsinki. All participants provided written informed consent.

### Image acquisitions and analysis

**Fig. 2:**
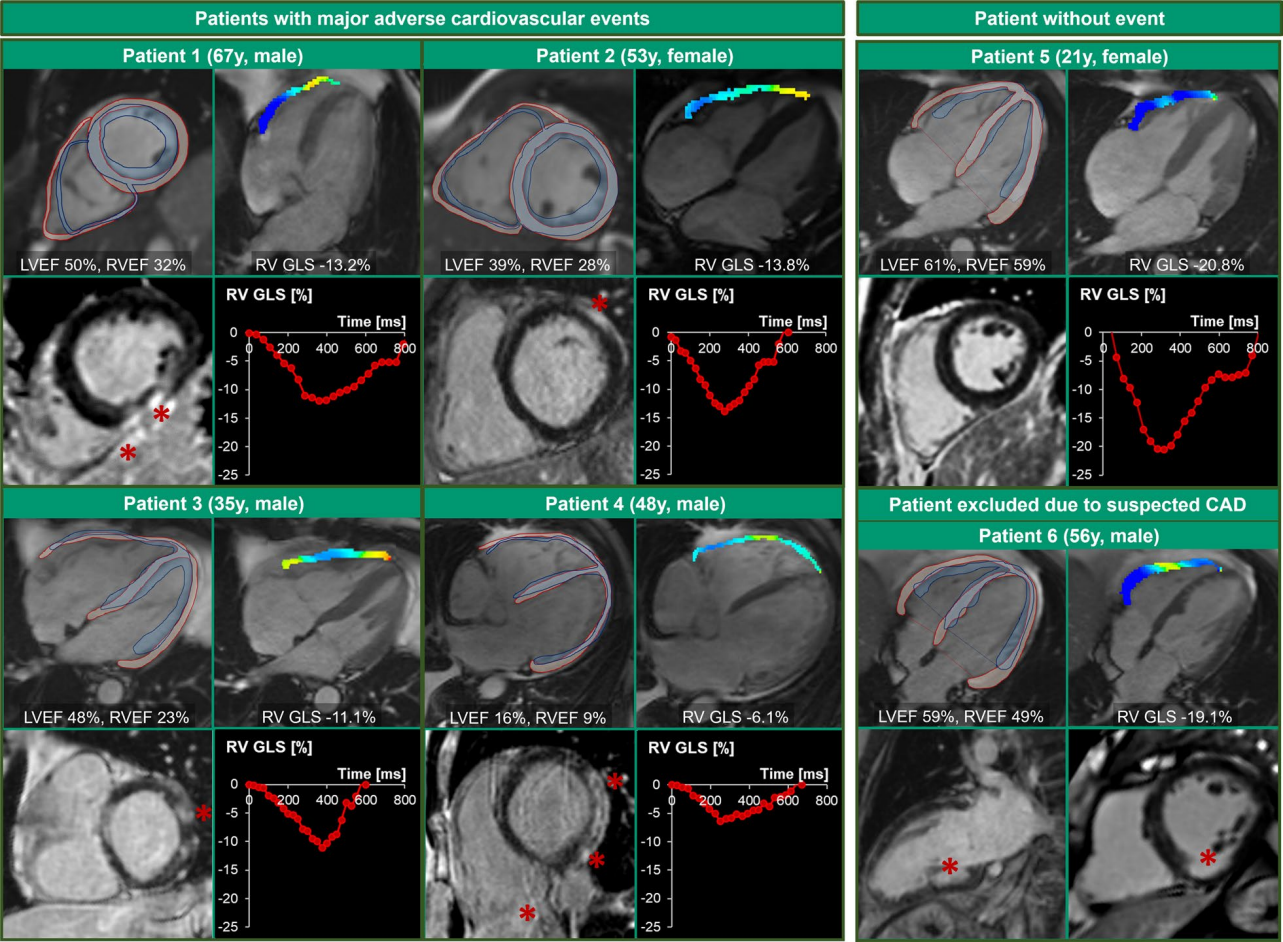
Examples of 6 patients referred to CMR for suspected myocarditis. LGE is marked with red asterisks. LGE images and short-axis slices were acquired in end-diastole (red contours). Blue contours represent the position of end-systolic contours. Strain overlays represent end-systolic peak strain, blue color mirrors more negative strain, while red color represents neutral or positive strain. CAD: coronary artery disease; EF: ejection fraction; GLS: global longitudinal strain; LV: left ventricle, RV: right ventricle

**Fig. 3:**
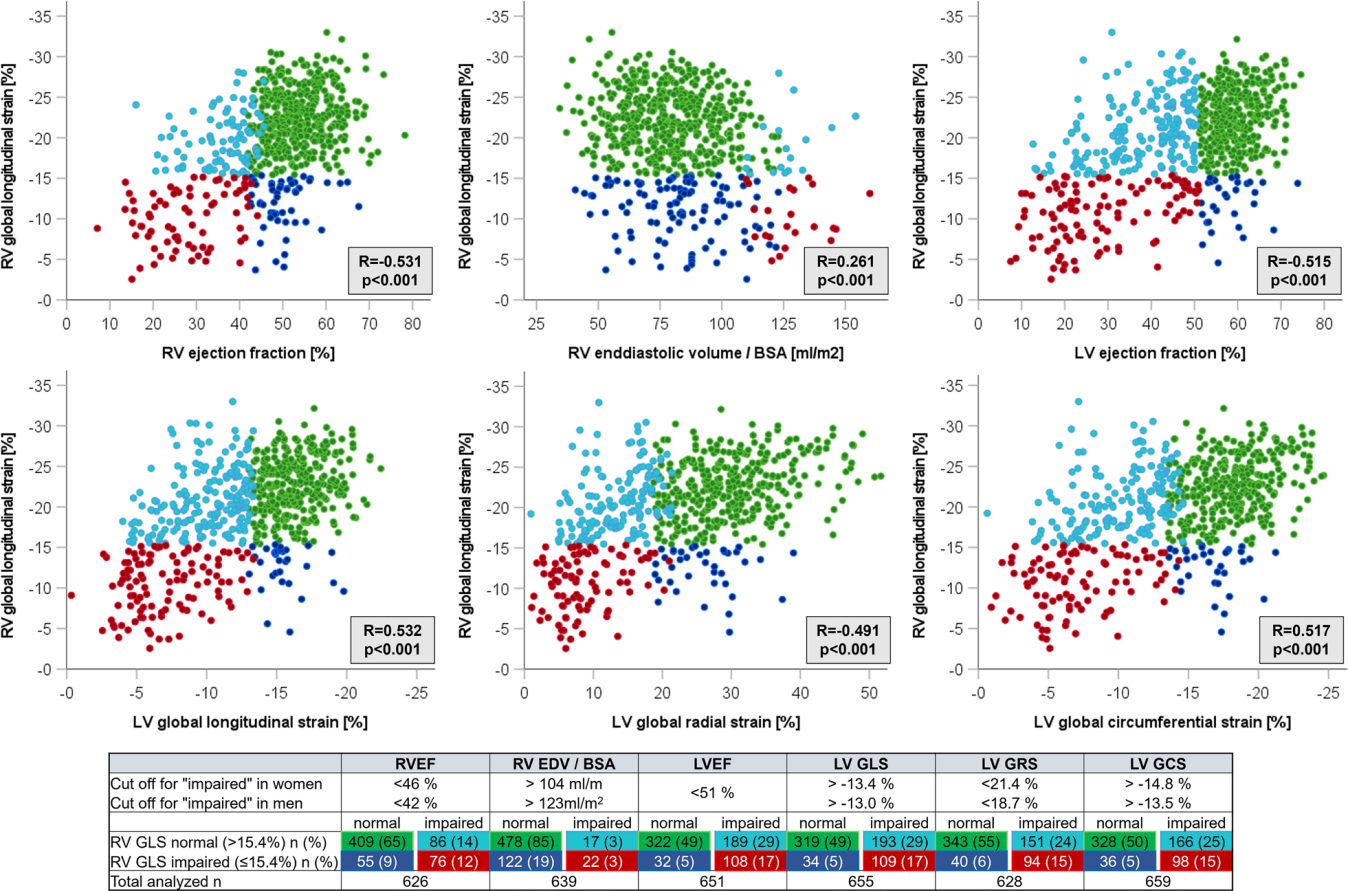
Correlation of right ventricular global longitudinal strain to other parameters of right ventricular and left ventricular function. Cut-off values were chosen according to [16–18]. BSA: body surface area; EF: ejection fraction; GLS/GRS/GCS: global longitudinal/radial/circumferential strain; EDV: end diastolic volume; LV: left ventricle, RV: right ventricle

CMR was performed at 1.5- or a 3.0 Tesla scanner (Magnetom Trio and Aera, Siemens Healthineers, Erlangen, Germany) as previously described [8, 9]. Patients received bodyweight-adjusted gadolinium (cumulative dose 0.1–0.2 mmol/kg). Post processing was performed with the CVI42 application (Circle Cardiovascular Imaging, Calgary, Canada, version 5.13.8) following standardized protocols in the core laboratory at Inselspital Bern. Investigators were blinded to clinical patient characteristics and outcomes. Feature tracking based RV GLS was assessed in cine images of a 4-chamber view (CV) after automatic and manually corrected tracing of the endo- and epicardial borders of the RV free wall without the RV septal wall (Fig. 2). LV GLS was averaged from assessments in the apical 2-, 3-, and 4-CV by tracing endo- and epicardial borders. Papillary muscles were included in the blood pool. End-systole and end-diastole were volumetrically defined. LGE was evaluated visually in each American Heart Association (AHA) segment [15] and quantified using the full-width-half-max (FWHM) approach at a short-axis stack. Cut-off values for females and males for LVEF (< 51%), RVEF (< 46% and < 42%), and RV end diastolic volume index (EDVi) (> 104 ml/m^2^ and > 123 ml/m^2^) were defined according to Kawel-Boehm et al. as the lower limit of the mean in healthy volunteers [16] (see Fig. 3). Cut-off values for feature tracking strain were determined from studies that also used CVI42. Cut-off for impaired RV GLS was defined at > − 15.4% independent of gender [17]. LV GLS was considered impaired if ≥ − 13.4 in women and ≥ − 13.0 in men [18]. Cut-off values for LV global radial strain (GRS) and LV global circumferential strain (GCS) were ≤ 21.4% and ≥ − 14.8% in females and ≤ 18.7% and in ≥ − 13.5% in males [18]. The presence of edema was assessed in T2-weighted images (signal intensity ratio of the myocardium to skeletal muscle ≥ 2 [19]) in both ventricles and in the LV also in T2 maps if available.

### Statistical analysis

Statistical analysis was performed with IBM SPSS Statistics 25 (IBM Corp., Armonk, New York, USA) and R software version 4.1.3 (R Foundation for Statistical Computing, Vienna, Austria). Results are presented as absolute frequencies (percentages) or as mean ± standard deviation (SD) whenever appropriate. Non-normally distributed variables were provided as the median with the corresponding interquartile range (IQR). Bivariate Pearson correlation was used to describe a correlation between RV GLS and other parameters of LV and RV function. Uni- and multivariate Cox regression was used to investigate associations to primary and secondary endpoints. The prognostic capability of stepwise models with sequentially included variables was evaluated by the goodness of fit, indicated by χ^2^ and compared to the subsequent model by a likelihood ratio test. Variables with > 10% missing values (e.g. biomarkers, results for LV or RV edema) were excluded. Time to event curves were plotted according to the Kaplan–Meier method. Results were considered to be statistically significant if the two-sided p-value was < 0.05.

## Results

1125 patients referred to CMR for suspected myocarditis were screened for eligibility. Of those, 75 patients were excluded due to known (medical records, invasive angiography) or suspected (according to CMR) coronary artery disease, 40 patients due to evidence of other structural cardiomyopathy and 4 patients due to known pulmonary artery hypertension. Among the remaining 941 patients, 162 did not meet ESC-criteria for clinically suspected myocarditis, in 114 RV strain analysis was not possible due to poor image quality, RV foreshortening, or breathing or arrhythmic artefacts and 6 (0.53%) were lost to follow-up. Finally, 659 patients (mean age 48.1 ± 16.1 years; 414 (62.8%) male) were included (Fig. 1) and underwent CMR imaging at median 10 (IQR 4–28) days after symptom onset. Clinical patient characteristics at baseline are presented in Table 1. LV function was impaired in 297 (45.6%) patients based on LVEF and in 302 (46.1%) based on LV GLS. Impaired LV function was more frequent in patients with impaired RV GLS (Table 2). RV function was impaired in 162 (25.9%) according to RVEF and in 144 (21.9%) patients according to RV GLS. Epicardial or transmural (originating from epicardial) LGE was present in 430 (65.3%) patients in the LV and 76 (11.5%) in the RV. Impaired RV GLS was significantly associated with the presence of RV LGE (p < 0.001) and RV edema (p = 0.042) but not LV LGE (p = 0.698) and LV edema (p = 0.125). Poor image quality or missing T2-weighted images or T2 maps did not allow the assessment of edema in 129 (19.6%) patients in the LV and in 159 patients (24.1%) in the RV. RV GLS was significantly correlated to LVEF, LV GLS, LV GCS and LV GRS in addition to moderate correlation to RVEF and weak correlation to the body-surfaced area (BSA)-indexed RV EDV (Fig. 3).

**Table 1:** Patient characteristics

	Overall	RV GLS abnormal	RV GLS normal	p value
		(> − 15.4%)	(≤ − 15.4%)	
	N = 659	N = 144	N = 515	
Demographical data				
Gender (male) n (%)	414 (62.8)	102 (70.8)	312 (60.6)	**0.024**
Age [years] mean ± SD	48.1 ± 16.1	49.5 ± 15.4	47.7 ± 16.3	0.240
BMI [kg/m^2^] mean ± SD	27.4 ± 5.9	28.4 ± 6.6	27.2 ± 5.7	**0.024**
BSA [m^2^] mean ± SD	2.0 ± 0.3	2.0 ± 0.3	2.0 ± 0.3	0.319
Myocarditis symptoms n (%)				
Chest pain	248 (37.8)	25 (17.4)	223 (43.3)	**< 0.001**
Arrhythmia^a^	124 (18.8)	25 (17.4)	99 (19.2)	0.613
Dyspnea NYHA II, III or IV	297 (45.1)	95 (67.4)	202 (39.2)	**< 0.001**
Time symptom onset to CMR [days] median (IQR)^c^	10 (4–28)	14 (7–28)	8 (4–28)	**0.038**
Biomarkers				
Troponin T [ng/l] median (IQR)	19 (1–115)	5 (1–28)	29 (1–198)	**< 0.001**
Above cutoff^b^ n (% of available^c^)	186 (55)	28 (37.8)	158 (59.8)	**0.001**
Creatine kinase [U/l] median (IQR)	180 (80–505)	135 (56–416)	198 (89–523)	0.072
Above cutoff^b^ n (% of available^c^)	159 (56.4)	27 (44.3)	132 (59.7)	**0.031**
ECG n (% of available^c^)				
Left bundle branch block	55 (9.2)	22 (16.9)	33 (7.1)	**0.001**
Right bundle branch block	37 (5.6)	7 (5.4)	30 (6.4)	0.673
ST-segment elevation	57 (8.6)	6 (4.6)	51 (10.9)	**0.029**

BMI: body-mass-index; BSA: body surface area; CMR: cardiovascular magnetic resonance; ECG: electrocardiogram; IQR: interquartile range; NYHA: New York Heart Association; RV GLS: right ventricular global longitudinal strain; SD: standard deviation

^a^Arrhythmia comprised ECG-documented sustained and non-sustained ventricular tachycardia

^b^Cutoff values for elevated troponin T and creatine kinase were time- and site-specific according to the essay used for analysis, and > 190U/l, respectively

^c^Date of symptom onset was available in 393 (59.6%) patients, troponin T was available in 338 (51.3%) patients, and creatine kinase in 282 (42.8%) patients. ECG was available in 598 (90.7%) patients

**Table 2:** Imaging findings

	Overall	RV GLS abnormal	RV GLS normal	p value
		(> − 15.4%)	(≤ − 15.4%)	
	N = 659	N = 144	N = 515	
Cardiac function mean ± SD				
LV EDV [ml]	192 ± 69.8	235.8 ± 91.6	180.6 ± 57.8	**< 0.001**
LV EDV/BSA [ml/m^2^]	98.7 ± 33.9	119.6 ± 45.1	93.2 ± 27.9	**< 0.001**
LV EF [%]	48.4 ± 15.1	35.7 ± 17	51.9 ± 12.5	**< 0.001**
LV EF**<** 51% n (%)	297 (45.6)	108 (77.1)	189 (37.0)	**< 0.001**
RV EDV [ml]	161.2 ± 49.8	180.6 ± 59.6	156.1 ± 45.6	**< 0.001**
RV EDV/BSA [ml/m^2^]	82.3 ± 21	90.6 ± 25.1	80.2 ± 19.3	**< 0.001**
RV EF [%]	47.9 ± 11.8	37.2 ± 13.3	50.7 ± 9.5	**< 0.001**
RV EF abnormal n (%)	162 (25.9)	76 (58.0)	86 (17.4)	**< 0.001**
Feature tracking mean ± SD				
LV GLS [%]	− 12.9 ± 4.5	− 9.0 ± 4.6	− 13.9 ± 3.8	**< 0.001**
LV GLS abnormal n (%)	302 (46.1)	109 (76.2)	193 (37.7)	**< 0.001**
LV GRS [%]	22.8 ± 10.9	13.34 ± 8.02	26.18 ± 9.86	**< 0.001**
LV GCS [%]	− 14.3 ± 5.3	− 9.7 ± 5.4	− 15.5 ± 4.6	**< 0.001**
RV time to peak longitudinal strain [ms]	310.4 ± 67.2	287.5 ± 89.8	316.7 ± 57.9	**< 0.001**
RV longitudinal strain rate [1/s]	− 1.21 ± 0.58	− 0.93 ± 0.49	− 1.29 ± 0.59	**< 0.001**
LGE				
LV LGE present n (%)	430 (65.3)	92 (63.9)	338 (65.6)	0.698
LGE involves insertion points n (%)	130 (19.7)	33 (22.9)	97 (18.8)	0.277
LGE septal n (%)	225 (34.2)	62 (43.1)	163 (31.7)	**0.011**
LGE number of AHA-segments mean ± SD	2.9 ± 3.2	3.31 ± 3.36	2.65 ± 3.06	0.741
LGE extent FWHM [g] mean ± SD	5.3 ± 8.5	4.6 ± 9.3	5.4 ± 8.4	0.090
LGE RV				
Not definable due to image quality	270 (41)	67 (46.5)	203 (39.4)	
LGE RV present n (%)	75 (11.4)	27 (18.8)	48 (9.3)	**< 0.001**
LGE RV absent n (%)	314 (47.5)	50 (34.7)	264 (51.3)	
LV edema n (% of available^a^)	204 (38.5)	35 (32.1)	169 (40.1)	0.125
RV edema n (% of available^a^)	131 (26.2)	40 (32.8)	91 (24.1)	**0.042**

AHA: American Heart Association; BSA: body surface area; EDV: end diastolic volume; EF: ejection fraction; FWHM: full width half max; GCS/GLS/GRS: global circumferential/longitudinal/radial strain; LGE, late gadolinium enhancement; LV: left ventricle; RV: right ventricle; SD: standard deviation

^a^Poor image quality or missing sequences (T2-weighted images or LV T2 maps) did not allow the assessment of edema in 129 (19.6%) patients in the LV and in 159 patients (24.1%) in the RV

During the observation time of median 3.7 (IQR 2.1 to 6.3) years, first MACE occurred in 115 patients (17.6%), of whom 45 (6.8%) patients were hospitalized due to HF, 42 (6.4%) patients died, 33 (5%) developed VT and 16 (2.4%) suffered from recurrent myocarditis. A total of 21 patients experienced more than one event type. Univariate Cox regression analysis demonstrated the significant association of female gender, elevated N-terminal pro-hormone brain natriuretic peptide (NT-proBNP), LV LGE and LV LGE extent, reduced LVEF and RVEF, LV strains and RV GLS to the combined endpoint (Table 3). In multivariable Cox regression analysis, *model 1a* including the imaging variables LGE extent, LV GLS and impaired LVEF and RVEF predicted MACE with a χ^2^ of 41.97 (p < 0.001). Implementing RV GLS in addition (*model 1b*) or instead of LV GLS (*model 1c*) did not significantly improve prognostication (χ^2^ = 51.65; p = 0.932 for *model 1b* and χ^2^ = 47.6; p = 0.623 for *model 1c*).

**Table 3:** Cox Regression models for the combined endpoint of major adverse cardiovascular events (MACE)

MACE	Univariable analyses		Multivariable model 1a Imaging parameter without RV GLS		Multivariable model 1b Imaging parameter with additional RV GLS		Multivariable model 1c Imaging parameter with RV GLS instead of LV GLS	
	HR (95% CI)	p value	HR_adjusted_ (95% CI)	p value	HR_adjusted_ (95% CI)	p value	HR_adjusted_ (95% CI)	p value
Clinical characteristics								
Age [years]	1.01 (1.00–1.03)	**0.022**						
Gender [male]	0.65 (0.45–0.94)	**0.023**						
Troponin T peak^a^ [ng/l]	0.99 (0.99–1.00)	0.206						
NT-proBNP^a^ [log pg/ml]	2.45 (1.54–3.90)	**< 0.001**						
CMR LGE								
LV LGE present	1.61 (1.08–2.32)	**0.024**						
LV LGE involves insertion points	1.22 (0.77–1.94)	0.390						
LV LGE extent FWHM [g]	1.04 (1.02–1.06)	**< 0.001**	1.03 (1.01–1.05)	**0.005**	1.03 (1.01–1.05)	**0.005**	1.03 (1.01–1.05)	**0.005**
RV LGE present^a^	1.86 (1.07–3.24)	**0.027**						
CMR ejection fraction								
LV EF [%]	0.96 (0.95–0.98)	**< 0.001**	1.00 (0.98–1.03)	0.726	1.00 (0.98–1.03)	0.728	0.98 (0.96–1.00)	0.074
RV EF [%]	0.95 (0.94–0.97)	**< 0.001**	0.98 (0.96–0.99)	**0.038**	0.98 (0.96–1.00)	0.072	0.98 (0.96–1.00)	0.082
CMR feature tracking								
LV GLS [%]	1.15 (1.10–1.20)	**< 0.001**	1.10 (1.01–1.20)	**0.037**	1.10 (1.01–1.21)	**0.044**		
LV GCS [%]	1.12 (1.08–1.16)	**< 0.001**						
LV GRS [%]	0.94 (0.92–0.96)	**< 0.001**						
RV GLS [%]	1.07 (1.04–1.10)	**< 0.001**			1.00 (0.96–1.04)	0.932	1.01 (0.97–1.05)	0.622
Model Chi-Square			41.97		51.65		47.6	
Df			4		5		4	
p value			**< 0.001**		**< 0.001**		**< 0.001**	
− 2 Log-Likelihood			1074.4		1074.4		1179.5	
p-value vs. model without RV GLS					0.932		0.623	

CI: confidence intervals; EF: ejection fraction; Df: degrees of freedom; FWHM: full width half max; GCS/GLS/GRS: global circumferential/longitudinal/radial strain; HR: Hazard ratio; LGE: late gadolinium enhancement; LV: left ventricle; NT-proBNP: N-terminal pro-hormone brain natriuretic peptide; RV: right ventricle

^a^Variables with > 20% missing values. Troponin was available in 338 (51.3%) patients. NT-proBNP was available in 159 (24.1%) of patients. RV LGE was not assessable in 270 (41%) of patients

Regarding the secondary endpoints, male sex, elevated NT-proBNP, LV LGE extent, impaired RV and LV ejection fraction and strain were associated to HF hospitalizations in univariate analysis (Table 4). Nested models with imaging parameters demonstrated the incremental prognostic value of LV GLS and RVEF (*model 2a*) as reported previously [5, 9]. The addition of RV GLS (*model 2b)* did not improve prognostication (χ^2^ = 85.2; p = 0.194 vs. *model 2a*). However, implementing RV GLS instead of LV GLS in the newly constructed *model 2c* achieved comparable prognostic capability, as reflected by a significant model χ^2^ of 80.8 (p = 0.048 vs. without RV GLS). RV GLS remained independently associated with the occurrence of HF hospitalizations, adjusted to the effects of the other variables in the model (HR_adjusted_ = 1.07, 95% CI 1.01–1.15; p = 0.048). Regarding the other secondary endpoints, RV GLS was univariately associated with death (HR = 1.07; 95% CI 1.02–1.12; p = 0.004), but not after adjustment for LVEF and RVEF or LV GLS (Table 5). RV GLS was not associated to VT (HR = 1.02; 95% CI 0.96–1.08; p = 0.509) or recurrent myocarditis (HR = 0.95; 95% CI 0.86–1.04; p = 0.258) (Table 6).

**Table 4:** Cox Regression models for the secondary endpoint of heart failure hospitalizations

Heart failure hospitalizations	Univariable analyses		Multivariable model 2a Imaging parameter without RV GLS		Multivariable model 2b Imaging parameter with additional RV GLS		Multivariable model 2c Imaging parameter with RV GLS instead LV GLS	
	HR (95% CI)	p value	HR_adjusted_ (95% CI)	p value	HR_adjusted_ (95% CI)	p value	HR_adjusted_ (95% CI)	p value
Clinical characteristics								
Age [years]	1.01 (0.99–1.03)	0.145						
Gender [male]	0.46 (0.26–0.83)	**0.010**						
Troponin T peak^a^ [ng/l]	1.00 (0.99–1.00)	0.356						
NT-proBNP^a^ [log pg/ml]	3.45 (1.70–6.90)	**< 0.001**						
CMR LGE								
LV LGE present	1.36 (0.71–2.60)	0.350						
LV LGE involves insertion points	0.75 (1.32–1.79)	0.525						
LV LGE extent FWHM [g]	1.05 (1.02–1.08)	**0.003**	1.03 (1.00–1.06)	0.061	1.03 (1.00–1.06)	0.064	1.03 (1.00–1.06)	0.063
RV LGE present^a^	2.33 (0.96–5.67)	0.062						
CMR ejection fraction								
LV EF [%]	0.93 (0.91–0.95)	**< 0.001**	1.00 (0.95–1.05)	0.962	1.00 (0.95–1.05)	0.943	0.96 (0.93–0.99)	**0.012**
RV EF [%]	0.91 (0.89–0.93)	**< 0.001**	0.96 (0.93–0.99)	**0.038**	0.97 (0.93–1.01)	0.179	0.97 (0.94–1.02)	0.226
CMR feature tracking								
LV GLS [%]	1.38 (1.28–1.50)	**< 0.001**	1.26 (1.07–1.48)	**0.007**	1.22 (1.02–1.44)	**0.025**		
LV GCS [%]	1.30 (1.21–1.39)	**< 0.001**						
LV GRS [%]	0.85 (0.81–0.89)	**< 0.001**						
RV GLS [%]	1.17 (1.12–1.23)	**< 0.001**			1.05 (0.98–1.13)	0.195	1.07 (1.01–1.15)	**0.048**
Model Chi-Square			62.5		85.2		80.8	
Df			4		5		4	
p value			**< 0.001**		**< 0.001**		**< 0.001**	
−2 Log-Likelihood			313.0		311.3		316.8	
p-value vs. model without RV GLS					0.194		**0.048**	

CI: confidence intervals; EF: ejection fraction; Df: degrees of freedom; FWHM: full width half max; GCS/GLS/GRS: global circumferential/longitudinal/radial strain; HR: Hazard ratio; LGE: late gadolinium enhancement; LV: left ventricle; NT-proBNP: N-terminal pro-hormone brain natriuretic peptide; RV: right ventricle

^a^Variables with > 20% missing values. Troponin was available in 338 (51.3%) patients. NT-proBNP was available in 159 (24.1%) of patients. RV LGE was not assessable in 270 (41%) of patients

**Table 5:** Cox Regression Models for the secondary endpoint of all-cause mortality

Death	Univariable analyses		Multivariable model 3a Imaging parameter without RV GLS		Multivariable model 3b Imaging parameter with additional RV GLS		Multivariable model 3c Imaging parameter with RV GLS instead LV GLS	
	HR (95% CI)	p value	HR_adjusted_ (95% CI)	p value	HR_adjusted_ (95% CI)	p value	HR_adjusted_ (95% CI)	p value
Clinical characteristics								
Age [years]	1.05 (1.03–1.07)	**< 0.001**						
Gender [male]	0.53 (0.29–0.97)	**0.040**						
Troponin peak^a^ [ng/l]	1.00 (0.99–1.00)	0.173						
NT-proBNP^a^ [log pg/ml]	5.01 (1.83–13.7)	**0.002**						
CMR LGE								
LV LGE present	1.22 (0.63–2.35)	0.551						
LV LGE involves insertion points	1.38 (0.66–2.92)	0.394						
LV LGE extent FWHM [g]	1.03 (0.99–1.07)	0.153						
RV LGE present^a^	1.41 (0.54–3.65)	0.481						
CMR ejection fraction								
LV EF [%]	0.97 (0.95–0.99)	**< 0.001**	1.00 (0.96–1.04)	0.951	1.00 (0.96–1.04)	0.935	0.98 (0.96–1.01)	0.283
RV EF [%]	0.96 (0.94–0.98)	**0.001**	0.98 (0.94–1.01)	0.215	0.98 (0.95–1.02)	0.346	0.98 (0.95–1.02)	0.348
CMR feature tracking								
LV GLS [%]	1.14 (1.06–1.21)	**< 0.001**	1.08 (0.93–1.24)	0.310	1.06 (0.92–1.23)	0.393		
LV GCS [%]	1.10 (1.04–1.17)	**0.001**						
LV GRS [%]	0.95 (0.92–0.98)	**0.001**						
RV GLS [%]	1.07 (1.02–1.12)	**0.004**			1.02 (0.96–1.09)	0.526	1.03 (0.96–1.09)	0.405
Model Chi-Square			15.2		15.9		14.9	
Df			3		4		3	
p value			**0.002**		**0.003**		**0.002**	
−2 Log-Likelihood			440.0		439.6		440.8	
p-value vs. model without RV GLS					0.528		0.408	

CI: confidence intervals; EF: ejection fraction; Df: degrees of freedom; FWHM: full width half max; GCS/GLS/GRS: global circumferential/longitudinal/radial strain; HR: Hazard ratio; LGE: late gadolinium enhancement; LV: left ventricle; NT-proBNP: N-terminal pro-hormone brain natriuretic peptide; RV: right ventricle

^a^Variables with > 20% missing values. Troponin was available in 338 (51.3%) patients. NT-proBNP was available in 159 (24.1%) of patients. RV LGE was not assessable in 270 (41%) of patients

**Table 6:** Univariable Cox Regression for the secondary endpoints of sustained VT and recurrent myocarditis

	Sustained VT Univariable analyses		Recurrent myocarditis Univariable analyses	
	HR (95% CI)	p value	HR (95% CI)	p value
Clinical characteristics				
Age [years]	1.00 (0.98–1.03)	0.742	0.94 (0.91–0.98)	**0.002**
Gender [male]	0.80 (0.40–1.59)	0.523	1.31 (0.46–3.78)	0.614
Troponin T peak^a^ [ng/l]	1.00 (0.99–1.00)	0.266	1.00 (1.00–1.00)	0.301
NT-proBNP^a^ [log pg/ml]	1.27 (0.54–2.99)	0.584	0.70 (0.20–2.46)	0.576
CMR LGE				
LV LGE present	2.47 (1.18–4.54)	**0.012**	2.30 (0.66–8.08)	0.194
LV LGE involves insertion points	1.50 (0.67–3.34)	0.320	0.70 (0.16–3.11)	0.641
LV LGE extent FWHM [g]	1.07 (1.05–1.10)	**< 0.001**	1.03 (0.97–1.09)	0.393
RV LGE present^a^	2.48 (0.96–6.42)	0.061	1.21 (0.32–4.58)	0.783
CMR ejection fraction				
LV EF [%]	0.96 (0.94–0.98)	**< 0.001**	1.07 (1.02–1.13)	**0.009**
RV EF [%]	0.95 (0.93–0.97)	**< 0.001**	1.04 (0.99–1.09)	0.106
CMR feature tracking				
LV GLS [%]	1.13 (1.05–1.21)	**0.002**	0.82 (0.71–0.95)	**0.009**
LV GCS [%]	1.13 (1.06–1.21)	**< 0.001**	0.86 (0.76–0.97)	**0.014**
LV GRS [%]	0.93 (0.90–0.97)	**< 0.001**	1.06 (1.01–1.11)	**0.017**
RV GLS [%]	1.02 (0.96–1.08)	0.509	0.95 (0.86–1.04)	0.258

CI: confidence intervals; EF: ejection fraction; FWHM: full width half max; GCS/GLS/GRS: global circumferential/longitudinal/radial strain; HR: Hazard ratio; LGE: late gadolinium enhancement; LV: left ventricle; NT-proBNP: N-terminal pro-hormone brain natriuretic peptide; RV: right ventricle; VT: ventricular tachycardia

^a^Variables with > 20% missing values. Troponin was available in 338 (51.3%) patients. NT-proBNP was available in 159 (24.1%) of patients. RV LGE was not assessable in 270 (41%) of patients

**Fig. 4:**
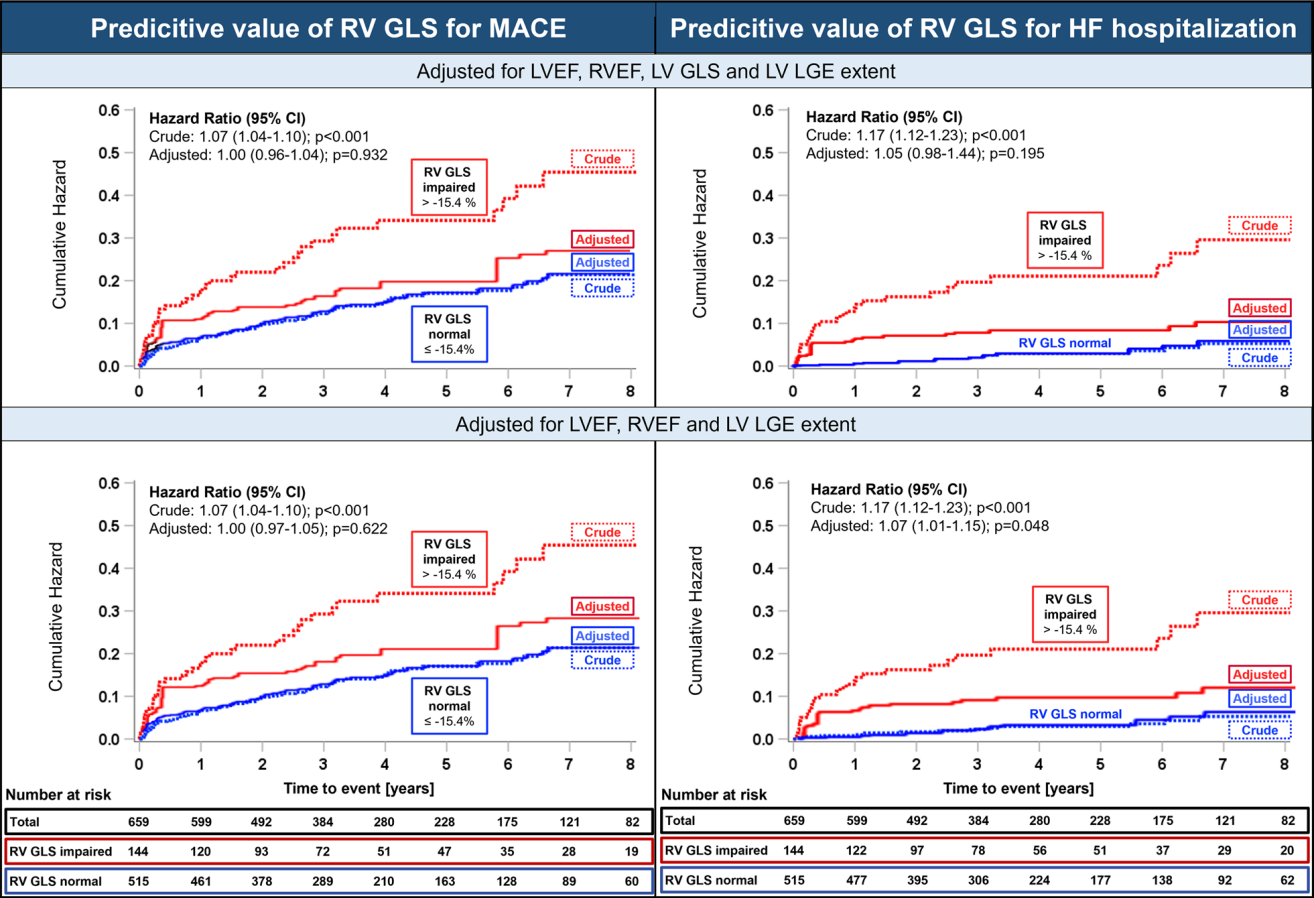
Crude and adjusted time to event-curves for the association of right ventricular global longitudinal strain and heart failure hospitalizations. CI: confidence intervals, RV GLS: right ventricular global longitudinal strain

The adjusted time to event curves for the combined endpoint of first MACE and for the secondary endpoint of HF hospitalizations are plotted in Fig. 4 after dichotomizing the cohort into patients with normal versus impaired RV GLS and adjustment for LGE extent, RVEF, LVEF (bottom row) and LV GLS (upper row). Baseline results stratified by the occurrence of first MACE are presented in Additional file 1: Table S1.

## Discussion

The present study demonstrated that nearly one quarter of patients with suspected myocarditis suffers from RV injury and impaired RV GLS. Impaired RV GLS was associated with the presence of LGE and edema in the RV, but not in the LV and linked to reduced LVEF and RVEF. RV GLS was strongly associated in the univariate analysis to cardiovascular outcomes in the clinical setting of suspected myocarditis but did not provide incremental prognostic value in the prediction of MACE after adjustment for RVEF, LVEF, LV GLS and LGE extent. Nevertheless, the study demonstrated that RV GLS may provide additional predictive value beyond RVEF, LVEF and LGE extent for HF hospitalizations in suspected myocarditis. This effect was comparable to the prognostic power of LV GLS in this setting, but not superior and restricted to HF hospitalizations as an endpoint. Therefore, RV GLS has only limited value in the clinical setting of suspected myocarditis.

Feature tracking based LV GLS analysis has proven its capability to refine risk-stratification across a large spectrum of cardiac diseases [20]. Recently, RV GLS has demonstrated prognostic value in right heart diseases such as arrhythmogenic cardiomyopathy but also dilated cardiomyopathy [21–23]. Both CMR parameters RV GLS and RVEF are highly robust, and reproducibility does not seem to limit their predictive power. Intraclass correlation coefficient for inter- and intra-reader variability for RV GLS ranged from 0.67 to 0.96 in prior studies [21, 24–26], in line with previously published results from our cohort (0.83 to 0.91) [8], and are comparable to the reproducibility of RVEF, ranging from 0.61 to 0.95 [27–29]. RV and LV GLS mirror different patterns of myocardial contractility compared to LVEF and RVEF which can be altered following different kind of injuries [30]. The volumetric measurement of EF is not exclusively determined by myocardial contractility but can be influenced by systemic or pulmonary artery blood pressure, volume status, valve disease, or obstructive disorders like pulmonary embolism. Changes in the end diastolic volume can lead to variations in EF independent of myocardial function and stroke volume [7]. LV strain measurements have been proven to overcome some of these limitations and inherit higher prognostic value than LVEF [9, 31], attributable to the potential to detect even subtle myocardial injury when LVEF is often preserved [9]. However, in this study, as well as in other reports [24, 32], more or a comparable number of patients suffered from impaired strain and impaired EF. This ratio might be partly explained by the selection of cut-off values, characteristics of the study cohort, post-processing software, and methodology across studies, while the high prognostic value of LV GLS was consistent across studies.

RV GLS is derived from a single slice (4CV) which makes it acquisition less time-intensive, but on the other hand also prone to impaired image quality. In our study, a relevant number of patients had to be excluded from RV based image analysis due to arrhythmia, breathing artefact, or foreshortening in the RV which limited the interpretation of our findings.

Improvement of RV image acquisition with dedicated RV focused views (i.e. carefully avoiding RV foreshortening in the 4-CH, dedicated 2-CH RV view, transaxial or transversal stacks) and RV adapted inversion time, could minimize the exclusion of images and help to exploit the additional information from RV tissue characteristics and RV function, including biplane RV GLS and radial or circumferential RV strain in future studies.

As previously discussed [5], impaired RV function and its association with adverse events in suspected myocarditis can result from primary inflammation of the RV myocardium, as a consequence of left sided HF and pulmonary hypertension or from pre-existing RV injury. In our study, impaired RV GLS was associated with RV LGE and RV edema, but not with LV LGE or LV edema, which is in line with prior reports that frequently observed dominant RV involvement in myocarditis in endocardial biopsy [33, 34]. Additionally, Luetkens et al. observed impaired RV GLS in patients with acute myocarditis that improved after recovery at 3 months compared to the baseline CMR [32], also pointing out that RV injury may directly occur from acute myocarditis and may lead to impaired RV GLS. On the other hand, RV function is of high prognostic relevance also in primary LV systolic dysfunction without RV inflammation [35], and due to the large number of inconclusive findings from the analysis of RV LGE and RV edema, our results cannot fully confirm or exclude a relationship between impaired RV function and acute RV inflammation. Pre-existing RV injury that might explain impaired RV GLS and its prognostic implications independent from the presence of myocarditis might be negligible in our study population because patients with known right heart disease by CMR findings or prior history were excluded. At baseline, patients with impaired RV GLS were more likely to exhibit HF features such as dyspnea and left-bundle branch block. In contrast, they less commonly presented with chest pain syndrome, elevated troponin levels, and ST-segment elevations in the ECG. We hypothesize that patients with primary LV involvement and associated chest pain syndrome, mimicking myocardial infarction, may have presented earlier in the course of the disease when troponin peaks and ST-elevations were still present. In contrast, patients with impaired RV GLS and dyspnea may have presented at later stages of myocarditis, coinciding with the onset of HF and a higher risk of future HF hospitalizations. This is further supported by our observation that patients with impaired RV GLS had a longer time gap between symptom onset and the conduction of CMR compared to those with normal RV GLS.

Regarding other imaging parameters, this study confirmed the high prognostic power of LV LGE and LV GLS in suspected myocarditis, which were both independently associated with MACE. The sole presence of LV LGE was associated with the composite of MACE and VT, but not with death, which contrasts prior studies [13, 36]. Myocardial scarring, indicated by LGE, can create a substrate for ventricular arrhythmias [37], but seems to be less important in the prediction of HF associated events following suspected myocarditis. Large extents of scarring however also can alter cardiac function, and trigger HF hospitalizations and mortality, evidenced by the observed association of LGE extent to HF hospitalizations. Consistently, Greulich et al. [13] observed an association between LGE and mortality in a cohort where LGE extent was larger, with LGE affecting an average of 4 segments. In our cohort, LGE extended over a mean of 2.9 segments, which may explain divagating findings regarding its association to mortality.

Our findings underline the important role of CMR in suspected myocarditis. CMR does not only allow to accurately assess LV dimension/function and tissue characteristics, but also proved to inherit important information from RV function. Echocardiography represents a reproducible method to derive RV GLS and RVEF but may be challenging in patients with impaired echo window [38], and RV GLS values only moderately agree to those of CMR, underlining the need for separate cut-off values [24, 39]. CMR-based evaluation of the updated Lake-Louise Criteria [40] and endomyocardial biopsy are currently the cornerstones in the diagnosis of myocarditis, also incorporating powerful outcome prognosticators from LV function and tissue characteristics [9, 11–13, 19, 41]. In patients without confirmed—, but clinically suspected myocarditis according to standardized criteria [1], feature tracking based strain analysis in the LV [9] may be helpful in the management of these patients, while—as demonstrated by this study—it is of limited value in the RV.

## Limitations

The retrospective design of this observational study inherits several limitations. The lack of standardized blood sampling protocols goes along with a relevant number of missing values for biomarkers such as NT-proBNP and troponin and their prognostic relevance could not be fully assessed. The prognostic implications of features that were incorporated in our inclusion criteria (e.g. sole presence of LGE or elevated biomarkers such as NT-proBNP) might be underestimated. Secondly, in order to cover the full spectrum of myocarditis, we included patients with clinically suspected myocarditis according to standardized criteria, which is one of the most common reasons for referral to CMR [42]. Nevertheless, we cannot fully exclude a selection bias since atypical right-sided myocarditis might not be fully reflected in the ESC-criteria and therefore less severe RV myocarditis might be underrepresented. Additionally, we did not conduct endomyocardial biopsy or genetic testing to exclude alternative diagnosis in all patients, which however represents current clinical practice among many centers [43]. The lack of systematic collection of endomyocardial biopsy samples and invasively assessed hemodynamic data precluded us from determining the etiology of RV dysfunction. Thirdly, we cannot exclude a bias resulting from the time gap of CMR imaging from symptom onset that did not follow predefined protocols and might have varied over time and across centers. The prevalence of features indicating acute inflammation such as edema or the amount of LGE might therefore be underestimated. The lack of follow-up imaging did not allow us to assess these changes over time. Fourthly, mortality was assessed by hospital reports, and telephone interviews with family member, and the causes of death cannot be provided. Fifthly, it is important to note that our study spanned a considerable period, from 2005 to 2019. Consequently, modern sequences like parametric mapping were not available for a significant number of scans and were not thoroughly explored in our study. Finally, our statistical analysis is partially based on dichotomization of the patient cohort into those with normal versus impaired RV GLS. The cutoff value was chosen according to the results of a relatively small sample of 100 patients at another center [17]. Site-specific reference values might go along with different findings.

## Conclusions

Right ventricular global longitudinal strain determined by CMR feature tracking is associated with first MACE, heart failure hospitalizations and death in the univariate analysis, but has neither independent nor incremental prognostic value after adjustment for LV/RV-function and tissue characteristics. Although RV GLS may provide some prognostic value for heart failure hospitalizations, its overall utility as a predictor of adverse cardiovascular events in the clinical setting of myocarditis remains limited.

### Supplementary Information

**Additional file 1: Table S1. **Baseline characteristics in patients with and without major adverse cardiovascular events (MACE).

## Data Availability

The datasets used and/or analyzed during the current study are available from the corresponding author on reasonable request.

## Abbreviations

AHA: American Heart Association
BMI: Body mass index
BSA: Body surface area
CAD: Coronary artery disease
CI: Confidence intervals
CMR: Cardiac magnetic resonance
CV: Chamber view
Df: Degrees of freedom
ECG: Electrocardiogram
EDV: End diastolic volume
EF: Ejection fraction
EMB: Endomyocardial biopsy
ESC: European Society of Cardiology
FWHM: Full width half max
GCS: Global circumferential strain
GLS: Global longitudinal strain
GRS: Global radial strain
HF: Heart failure
IQR: Interquartile range
LGE: Late gadolinium enhancement
LV: Left ventricle
MACE: Major adverse cardiovascular events
NT-proBNP: N-terminal pro-hormone brain natriuretic peptide
NYHA: New York Heart Association
RV: Right ventricle
SD: Standard deviation
VT: Ventricular tachycardia
