# Supplementary material for: Predictive value of cardiac magnetic resonance right ventricular longitudinal strain in patients with suspected myocarditis
Source: J Cardiovasc Magn Reson. 2023 Aug 17;25:49. doi: 10.1186/s12968-023-00957-6 (PMC10433613; doi:10.1186/s12968-023-00957-6)
Supplement: Supplementary file 1 — Additional file 1: Table S1. Baseline characteristics in patients with and without major adverse cardiovascular events (MACE). [file 12968_2023_957_MOESM1_ESM.docx]

**Additional file**

|  | **Overall** | **MACE** | **No MACE** |
| --- | --- | --- | --- |
|  | N = 659 | N = 115 | N = 544 |
| **Demographical data** |  |  |  |
| Gender (male) n (%) | 414 (62.8) | 61 (53) | 353 (64.9) |
| Age [years] mean ± SD | 48.1 ± 16.1 | 50.3 ± 16.6 | 47.8 ± 16 |
| BMI [kg/m^2^] mean ± SD | 27.4 ± 5.9 | 29.1 ± 7.6 | 27.1 ± 5.4 |
| **Biomarkers** |  |  |  |
| Troponin T [ng/l] median (IQR) | 19 (1-115) | 22 (1 to 157) | 12 (0 to 91) |
| above cutoff* n (% of available^$^) | 186 (55) | 34 (48.6) | 150 (55.9) |
| Creatine-Kinase [U/l] median (IQR) | 180 (80-505) | 182 (80 to 532) | 167 (73 to 408) |
| above cutoff* n (% of available^$^) | 159 (56.4) | 31 (52.5) | 128 (57.4) |
| **ECG n (% of available**^$^**)** |  |  |  |
| Left bundle branch block | 55 (9.2) | 12 (11.6) | 43 (8.7) |
| Right bundle branch block | 37 (5.6) | 7 (6.8) | 30 (6.1) |
| ST-segment elevation | 57 (8.6) | 8 (7.8) | 49 (9.9) |
| **Cardiac function** mean ± SD |  |  |  |
| LV EDV/BSA [ml/m^2^] | 98.7 ± 33.9 | 109.2 ± 37.9 | 96.6 ± 32.7 |
| LV EF [%] | 48.4 ± 15.1 | 40.6 ± 16.3 | 50.0 ± 14.3 |
| RV EDV/BSA [ml/m^2^] | 82.3 ± 21 | 84.6 ± 23.5 | 81.9 ± 20.5 |
| RV EF [%] | 47.9 ± 11.8 | 41.7 ± 14.5 | 49.1 ± 10.7 |
| **Feature tracking** mean ± SD |  |  |  |
| LV GLS [%] | -12.9 ± 4.5 | -10.4 ± 4.6 | -13.4 ± 4.2 |
| LV GRS [%] | 22.8 ± 10.9 | 16.8 ± 10 | 24 ± 10.7 |
| LV GCS [%] | -14.3 ± 5.3 | -11.2 ± 5.3 | -14.9 ± 5.1 |
| RV GLS [%] | -19.6 ± 5.9 | -17.6 ± 7.1 | -20 ± 5.6 |
| **LGE** |  |  |  |
| LV LGE present n (%) | 430 (65.3) | 82 (71.3) | 348 (64) |
| LGE involves insertion points n (%) | 130 (19.7) | 23 (20) | 107 (19.7) |
| LGE septal n (%) | 225 (34.2) | 52 (45.2) | 173 (31.8) |
| LGE extent FWHM [g] mean ± SD | 5.3 ± 8.5 | 6.6 ± 9.4 | 4 ± 5.8 |
| LGE RV present n (%) | 75 (11.4) | 19 (16.5) | 56 (10.3) |
| **LV edema** n (% of available^$^) | 204 (38.5) | 48 (41.7) | 156 (35.4) |
| **RV edema** n (% of available^$^) | 131 (26.2) | 31 (33.0) | 100 (24.6) |

**Supplemental Table S1: Baseline characteristics in patients with and without major adverse cardiovascular events (MACE)**

Abbreviations: BMI, body-mass-index; BSA, body surface area; CMR, cardiovascular magnetic resonance; ECG, electrocardiogram; EDV, end diastolic volume; EF, ejection fraction; FWHM, full width half max; GCS/GLS/GRS, global circumferential/longitudinal/radial strain; IQR, interquartile range; MACE, major adverse cardiovascular events; NYHA, New York Heart Association; LGE, late gadolinium enhancement; LV, left ventricle; RV, right ventricle; SD, standard deviation.

^§^Arrhythmia comprised ECG-documented sustained and non-sustained ventricular tachycardia

^*^Cutoff values for elevated Troponin T and Creatine-Kinase were time- and site-specific according to the essay used for analysis, and >190U/l, respectively.

^$^ Troponin was available in 338 (51.3%) patients, and Creatine-Kinase in 282 (42.8%) patients. ECG was available in 598 (90.7%) patients. Poor image quality or missing sequences (T2-weighted images or LV T2 maps) did not allow the assessment of edema in 129 (19.6%) patients in the LV and in 159 patients (24.1%) in the RV.
